# Supra-Short Ultrasound Protocol for Rotator Cuff Tears in the Emergency Department: Pilot Study

**DOI:** 10.5811/westjem.46984

**Published:** 2025-09-25

**Authors:** Tony Zitek, Robert A. Farrow, Michael Shalaby, Daniel Puebla, Alejandro Sanoja, Edward Lopez, Joseph McShannic, Yonghoon Lee, Nicole Warren, Daniella Lamour, Jiodany Perez, Michael Rosselli

**Affiliations:** * Mount Sinai Medical Center, Department of Emergency Medicine, Miami Beach, Florida; † Kaiser Permanente Modesto Medical Center, Department of Emergency Medicine, Modesto, California

## Abstract

**Introduction:**

Although ultrasound is readily available to emergency physicians and known to be very accurate for diagnosing rotator cuff tears, it is rarely used for this purpose. Our goal in this study was to develop and preliminarily assess the accuracy of a simplified shoulder ultrasound protocol (the “supra-short” protocol), designed to be used by emergency physicians for diagnosis of supraspinatus tears.

**Methods:**

We performed a pilot diagnostic accuracy study in which we assessed the accuracy of the supra-short protocol as performed by minimally trained emergency physicians for identifying supraspinatus tears in volunteers. As a criterion standard, a sports medicine physician also performed a complete shoulder ultrasound on each volunteer. We determined the test characteristics of the supra-short protocol for supraspinatus tears, as well as the median time to complete a scan and the percentage of images deemed adequate by expert review.

**Results:**

Nine emergency physicians performed a total of 40 bilateral supra-short scans on six volunteers (two of whom were known to have shoulder pathology and four of whom had normal shoulders). Of the 80 shoulders scanned, there were 18 cases in which complete ultrasound performed by the sports medicine physician revealed a supraspinatus tear; 12 (66.7%) of those were identified by the novice sonographers using the supra-short protocol. Overall, the sensitivity of the supra-short protocol was 66.7% (95% CI 29.9–92.5%) and the specificity was 87.1% (95% CI 70.2–96.4%). The median time to completion of each shoulder was 133 seconds (interquartile range 88–182). Upon expert image review, 80.0% of the images were deemed adequate.

**Conclusion:**

After minimal training, emergency physicians were able to quickly perform the supra-short US protocol but were only able to identify supraspinatus tears with moderate accuracy, suggesting the need for more extensive training before clinical use.

## INTRODUCTION

In 2021, over 1.47 million patients presented to an emergency department (ED) in the United States for a shoulder injury,^1^ and many others presented for atraumatic shoulder pain. Emergency physicians often order a radiograph for patients with shoulder pain, which is the recommended first-line test for both traumatic and atraumatic shoulder pain by the American College of Radiology.^2^,^3^ While radiographs are highly useful for identifying fractures and other bony pathology, they have limited value for the diagnosis of soft tissue pathology.^2^,^3^ When radiographs do not reveal the etiology of the shoulder pain, magnetic resonance imaging (MRI) is the recommended test for soft tissue pathology of the shoulder.^2^,^3^ However, given its high cost and limited availability, it is rarely used in the ED. Thus, patients who present to the ED often receive a non-specific diagnosis (such as “shoulder pain”), which they may justifiably feel is inadequate.

While MRI is expensive and often unavailable in the ED, point-of-care ultrasound (POCUS) is readily available and inexpensive. Although partial, rotator cuff tendon tears may be difficult to accurately diagnose on ultrasound,^4^ for complete tears, radiology,^5^ sports medicine,^6^ and orthopedic literature^7^ have all reported that MRI and ultrasound have comparable test characteristics. We believe that emergency physicians should try to diagnose complete rotator cuff tears to expedite referral to orthopedics for a potentially surgical problem.

Previous data have shown emergency physician-performed POCUS to be highly accurate for shoulder dislocations and fractures,^8^,^9^ but there have been no published studies assessing the accuracy of emergency physician-performed shoulder POCUS for soft tissue injuries. The published evidence is limited to a case series describing the use of POCUS for identifying rotator cuff pathology in four patients.^10^ Moreover, although the American College of Emergency Physicians lists musculoskeletal ultrasound as one of its core applications,^11^ there is no established protocol for soft tissue shoulder POCUS with regard to what structures should be assessed and how.

Given the potential of POCUS to substantially increase the diagnostic accuracy of emergency physicians assessing patients with shoulder pain along with the lack of data in this area, we developed a POCUS protocol for emergency physicians to evaluate the rotator cuff (called the “supra-short” protocol). We then performed a pilot study to assess the feasibility and accuracy of our POCUS protocol for diagnosing rotator cuff (specifically supraspinatus) tears.

## METHODS

### Study Design and Setting

We performed a pilot, diagnostic-accuracy study to preliminarily assess a novel POCUS protocol designed to be used by emergency physicians without fellowship training in sports medicine or ultrasound to diagnose supraspinatus tears. The study was performed on volunteers (not patients) who were recruited by two of the investigators from a pool of colleagues, friends, and family, some of whom had known shoulder pathology. Anyone (with or without shoulder pain) could volunteer. Volunteers signed written, informed consent and were not compensated. The study was approved by Mount Sinai Medical Center Institutional Review Board. We followed the Standards for Reporting of Diagnostic Accuracy Studies guidelines.

**Population Health Research Capsule**

What do we already know about this issue?

*Ultrasound is highly accurate for the diagnosis of rotator cuff tears but is rarely used for this purpose in the emergency department.*

What was the research question?

*Can emergency physicians use an abbreviated shoulder ultrasound protocol to accurately diagnose supraspinatus tears?*

What was the major finding of the study?

*The sensitivity was 66.7% (95% CI 29.9–92.5%), and the specificity was 87.1% (95% CI 70.2–96.4%).*

How does this improve population health?

*With further training, emergency physicians may be able to use this shoulder ultrasound protocol to rapidly identify and refer patients with rotator cuff tears.*

### Rationale and Development of the Supra-short Protocol

The supra-short protocol was designed by one attending physician in emergency medicine (EM) (who had no ultrasound- or sports medicine-fellowship training) and two emergency physicians with sports medicine- fellowship training. In the process of developing the supra-short protocol, they considered several issues. First, a complete shoulder ultrasound protocol generally requires the patient to place their arm in five different positions, and it includes an assessment (multiple views) of each of the following structures: long head of the biceps tendon; subscapularis tendon; coracoacromial ligament; supraspinatus tendon; infraspinatus tendon; teres minor tendon; the posterior glenohumeral joint recess; and the acromioclavicular joint. It can also involve a subacromial impingement test.^12^ The investigators involved in the development of the supra-short protocol believed that a complete shoulder ultrasound assesses for pathology that is not relevant to EM and that it is too cumbersome for emergency physicians without fellowship training.

Additionally, they felt that ED patients with acute shoulder pain would be unable to place their arm in the five different positions required for a complete shoulder ultrasound, with the modified Crass position being especially difficult.^13^ They also felt that emergency physicians would still likely obtain shoulder radiographs on ED patients who present with shoulder pain; thus, an assessment of bony pathology with POCUS would be duplicative.

Considering the above issues, the goal was to create an ultrasound protocol that was easy to learn, quick to perform, and high yield with regard to pathology seen in the ED. We focused only on the most common soft tissue injury of relevance to EM—rotator cuff tears. We focused on the supraspinatus tendon because supraspinatus tears are the most common rotator cuff tears.^14^ Indeed, a prior systematic review reported that traumatic rotator cuff tears involve the supraspinatus in 84% of cases.^15^ Ultimately, the supra-short protocol was designed to be used in conjunction with a shoulder radiograph to help identify patients with complete supraspinatus tears who might benefit from more rapid outpatient follow-up with sports medicine or orthopedic surgery.

### Description of the Supra-short Protocol

**Figure 1:**
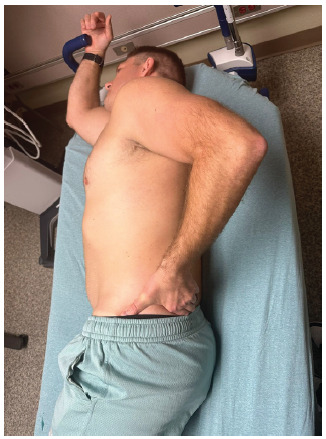
Volunteer in modified Crass position for the supra-short ultrasound protocol.

First, the patient is placed in the lateral decubitus position with the affected shoulder up and with the hand of the side of the affected shoulder on their posterior hip (also known as the modified Crass position. (Figure 1).

**Figure 2:**
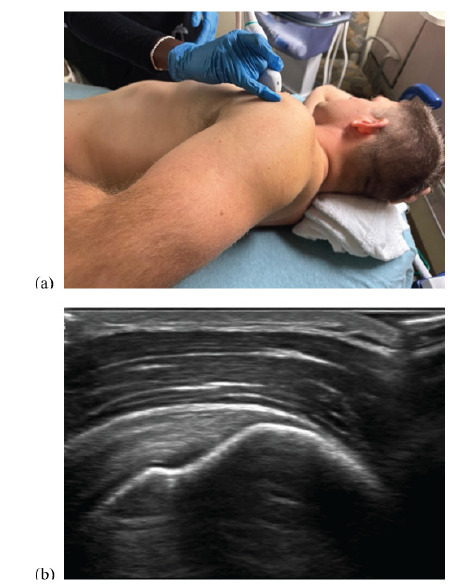
(a) Positioning of the ultrasound probe to obtain the long-axis view of the supraspinatus tendon using the supra-short protocol; and (b) sonographic long-axis view of a normal supraspinatus tendon.

Next, the sonographer places a high-frequency linear probe over the anterior shoulder, aligning the probe marker to point directly away from the umbilicus. In the correct location, this will provide a longitudinal view of the supraspinatus tendon (Figure 2).

**Figure 3:**
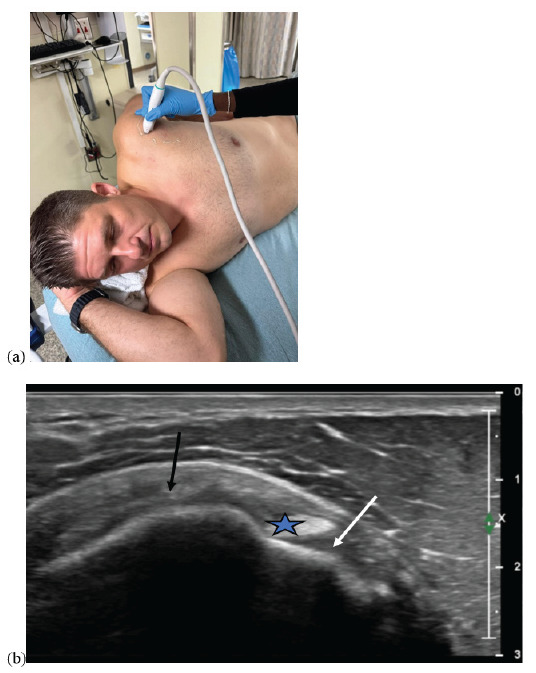
(a) Positioning of the ultrasound probe to obtain the transverse axis of the supraspinatus tendon using the supra-short protocol; and (b) normal transverse view of the supraspinatus tendon (black arrow). On this view, the biceps tendon (star) and subscapularis tendon (white arrow) are also visible (although not specifically assessed in the supra-short protocol).

Lastly, the ultrasound probe should be rotated toward the ear. This will provide a transverse view of the supraspinatus tendon (Figure 3). Although other pathology may be identified, the primary objective of the supra-short protocol is to identify a tear of the supraspinatus tendon, characterized by a visible discontinuity.

### Sonographers and Training

The principal investigator sent an email to EM attendings, fellows, and residents in search of volunteers to learn the supra-short protocol and apply it to a second group of volunteers. Nine physicians agreed to participate: four EM residents (one postgraduate year [PGY] 3, two PGY 2, and one PGY 1); three ultrasound fellows, and two non-fellowship trained attendings. The four residents and two attendings had minimal to no prior experience with shoulder ultrasound. The three ultrasound fellows had received some training as part of their fellowship curriculum, but none had ever used POCUS to diagnose a rotator cuff tear in a clinical setting.

The nine volunteer sonographers underwent a one-hour training session about normal supraspinatus anatomy, the supra-short protocol, and rotator cuff pathology. The training included a lecture with example images of supraspinatus tears and hands-on training on healthy volunteers. The training was led by an attending physician in EM with fellowship training in sports medicine and an EM attending physician with fellowship training in emergency ultrasound and extensive experience in musculoskeletal ultrasound.

### Data Collection

Nine days after their training session, all data were collected. We recruited six volunteer “patients,” two of whom had shoulder pain and four of whom did not. One volunteer had a recent MRI that demonstrated a complete supraspinatus tear of the left shoulder. The other volunteer with shoulder pain had not had an ultrasound or MRI prior to the day of data collection. A complete shoulder ultrasound performed by a sports medicine physician demonstrated this patient to have calcific tendinosis. The sonographers were blinded from the patient’s clinical history and performed no examination of the patient except for the supra-short ultrasound. Our initial plan was for all nine sonographers to perform the supra-short protocol on both shoulders of all six volunteers. However, variations in availability prevented every sonographer from scanning every volunteer (see Table 1).

A minimum of two images (one transverse and one longitudinal view of the supraspinatus) were saved for each shoulder. The supra-short ultrasounds were performed in the presence of an unbiased research staff member who timed each ultrasound. The start of each ultrasound was defined as the time when the sonographer’s hand first touched the probe, and the end of the ultrasound was defined as the time when the last image was saved. The research staff member asked the sonographer whether each shoulder had a supraspinatus tear (yes or no). Data from each ultrasound were recorded onto a paper data collection form. Sonographers were blinded to the scan results from earlier operators. Sonographers were not provided any information about what percentage of volunteers were expected to have pathology.

As a criterion standard, one practicing sports medicine physician performed complete shoulder ultrasounds on both shoulders of all six volunteers. All ultrasounds were performed using a 15 MHz linear transducer of a Philips Affiniti 50 ultrasound machine (Philips Healthcare, Amsterdam, the Netherlands).

### Ultrasound Image Review

After data collection, an EM attending with sports medicine-fellowship training reviewed all the images from the supra-short scans. This attending deemed whether each view was adequate to assess for the presence of a tear. If more than one image of a certain view was obtained (such as two transverse views of the supraspinatus), they reviewed both images and deemed the view adequate if either image was adequate. For images deemed inadequate, the reason for inadequacy was recorded.

### Outcomes

Our primary outcome was the accuracy (sensitivity and specificity) of the supra-short protocol for supraspinatus tears. Secondarily, we calculated the median time to perform a supra-short ultrasound and the percentage of ultrasound images that were deemed adequate. While we used all ultrasound scans (from each shoulder of each volunteer) for the estimates of the secondary outcomes, only the right shoulder of each volunteer was used for the calculation of the test characteristics. This was done because each shoulder is not independent from the other; thus, calculation of test characteristics using each shoulder as a separate test would not have been statistically valid. Sonographers were unaware that the right shoulder would be used for calculation of test characteristics.

### Data Analysis

In performing this pilot study, our goal was to preliminarily assess the supra-short protocol to determine whether adjustments to the protocol would be beneficial before performing a larger study on patients in a clinical setting. Therefore, we did not perform a formal power analysis to determine the sample size. One research assistant tabulated the data from the data collection forms into Excel v16.90.2 (Microsoft Corporation, Redmond, WA). Using the sports medicine fellowship-trained physician’s complete shoulder ultrasound as the criterion standard, we calculated the sensitivity and specificity (with 95% CIs) of the supra-short protocol for supraspinatus tear using the right shoulder (only) of each volunteer “patient.” Using data from the scan from both shoulders of each volunteer, we calculated the median (interquartile range [IQR]) duration of each supra-short ultrasound. Lastly, we calculated the percentage of ultrasound images deemed adequate.

## RESULTS

**Figure 4:**
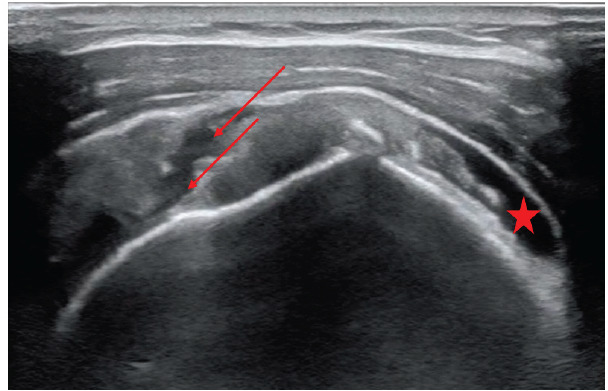
Long-axis view of the right supraspinatus tendon demonstrating an acute full thickness tear indicated by the red arrows with associated fluid collection. Also visible is a fluid collection superiorly (red star).

The nine emergency physicians performed a total of 40 bilateral supra-short ultrasound scans on six volunteers. Four volunteers were female, and two were male; they ranged in age from 25–62 years old. The complete ultrasound performed by the sports medicine physician revealed that one volunteer had bilateral supraspinatus tears (only the left shoulder had been known to have a tear prior to that date) and none of the other five volunteers had a supraspinatus tear. The volunteer with bilateral tears was scanned by all nine sonographers, which produced 18 shoulder views with a supraspinatus tear. Of those 18 cases, the sonographers using the supra-short protocol correctly identified the tear in 12 (66.7%). The supraspinatus tear from the right shoulder is shown in Figure 4.

The second volunteer “patient” who had shoulder pain was found to have pathology consistent with calcific tendinosis on complete ultrasound by the sports medicine physician. Four sonographers scanned this patient (for a total of eight shoulder scans). In one of the four cases of the abnormal shoulder, the sonographer interpreted the calcific tendonosis as a partial supraspinatus tear. In the other three cases, the sonographer using the supra-short protocol correctly deemed there to be no supraspinatus tear. The four volunteers who had no shoulder pain had normal complete shoulder ultrasounds; however, in those volunteers, there were six cases in which the sonographer thought there was a supraspinatus tear.

Overall, the sensitivity of the supra-short protocol in this study was 66.7% (95% CI 41.0–86.7%), the specificity was 88.7% (95% CI 78.1–95.3%), and the accuracy was 83.8% (95% CI 73.8–91.1%). Results stratified by level of training are shown in Table 2.

The median time to complete a supra-short ultrasound was 133 seconds (IQR 88–182 seconds). Expert review deemed 80.0 % of the images to be adequate.

## DISCUSSION

In this pilot study, we developed the supra-short ultrasound protocol, a novel POCUS technique designed to be used by emergency physicians without expertise in musculoskeletal ultrasound in conjunction with a radiograph to improve diagnostic accuracy in patients with shoulder pain. After one hour of training, a group of emergency physicians who were not experts in musculoskeletal ultrasound were able to obtain adequate views of the supraspinatus 80% of the time, and the median time to complete a scan was short—133 seconds. However, the sonographers were only moderately accurate in their ability to identify supraspinatus tears. In particular, EM residents (who were less experienced in the use of POCUS in general) were the least successful in identifying supraspinatus tears. These results suggest further training would be required before the supra-short protocol could be used diagnostically in the ED.

Despite the somewhat disappointing accuracy of the supra-short protocol in our study, we consider our results encouraging when accounting for the following statements. Currently, most emergency physicians rely on radiograph and physical exam maneuvers to make a diagnosis in patients with shoulder pain. Those assessments are unlikely to lead to an accurate diagnosis of a rotator cuff tear. In particular, in expert hands, the “full can test” and the “empty can test” were found to be 75% and 70% accurate, respectively, for supraspinatus tears (lower than the accuracy of the supra-short protocol in the hands of novices).^16^ Additionally, our sensitivity may have been lower than expected because the volunteer “patient” we recruited with a known supraspinatus tear was found to actually have bilateral supraspinatus tears. In musculoskeletal ultrasound, the sonographer is encouraged to compare the affected side to the contralateral (normal) side to help determine whether there is an abnormality.^17^ This strategy was of limited use in this volunteer, as both shoulders were affected.

Some emergency physicians may believe that POCUS to assess for rotator cuff tears is unnecessary, as they might suggest that all patients follow up for an MRI or with a specialist regardless of the results. However, we would argue that a patient with a supraspinatus tear seen on ultrasound often warrants more urgent outpatient follow-up with orthopedic surgery while a patient with a negative supra-short scan (and no other concerning findings) could follow up first with primary care or a non-surgical specialist. This protocol might be especially useful in resource-limited settings.

Lastly, an additional goal of this study was to determine whether our abbreviated shoulder POCUS protocol should be adjusted. Given that the accuracy of the protocol was only moderate in a group of individuals who were motivated to learn it, we do not think that adding additional views of the shoulder would have been beneficial. We envision that the supra-short protocol could be the basic, soft tissue shoulder ultrasound technique for emergency physicians analogous to assessment for pericardial effusion with the subxiphoid view of the heart. Those with additional interest or training could learn additional views of the shoulder in the same way that some emergency physicians learn more advanced cardiac POCUS skills.

## LIMITATIONS

The first limitation to our study is that we likely under-trained our sonographers. We hoped that by substantially simplifying the shoulder ultrasound to just two views we would be able to achieve high accuracy for supraspinatus tears with minimal training, but our data suggest that correct identification of supraspinatus tears requires more extensive training. Additionally, we performed this study on volunteers (rather than actual ED patients), which may have affected our results in multiple ways. For example, as mentioned above, the volunteer with a known supraspinatus tear ended up having bilateral tears, which likely made the diagnosis more difficult than it would be in a patient with an acute (unilateral) shoulder injury. On the other hand, four of the six volunteers had no shoulder pain at all, which might have made it easier to identify that they had no supraspinatus tear. However, given that sonographers were blinded to the patient’s symptoms and that we had six false positives on these volunteers, we do not think that inclusion of asymptomatic volunteers would have substantially boosted our test characteristics.

Another limitation to consider is that not all sonographers scanned all volunteers; this may have affected our estimates of test characteristics given the expected variability in ultrasound skill of the sonographers and challenges in scanning different volunteers. Finally, even if a supra-short ultrasound is completed and interpreted correctly, it will not identify all rotator cuff tears since it only assesses the supraspinatus. However, considering that the supraspinatus is involved in most rotator cuff tears, the supra-short protocol still has the potential to substantially improve diagnostic accuracy of rotator cuff tears in the ED.

## CONCLUSION

We developed a novel point-of-care ultrasound technique (the supra-short protocol) designed to be used by emergency physicians without expertise in musculoskeletal ultrasound to assess for rotator cuff (in particular, supraspinatus) tears. After minimal training, the emergency physicians learned how to obtain the views of the supra-short protocol and performed the scans quickly. They were only able to identify supraspinatus tears with moderate accuracy; thus, more extensive training on identifying pathology would be needed before this protocol could be used diagnostically. Nonetheless, the supra-short protocol may provide a means by which a non-expert in musculoskeletal US may assess for rotator cuff tears; further study is warranted.

## Figures and Tables

**Table 1:** The number of sonographers who scanned each volunteer using the supra-short ultrasound protocol.

Volunteer	Pathology	Number of sonographers completing scans (by level of training)		

		US Fellow	Attending	Resident
1	Bilateral supraspinatus tears	3	2	4
2	Right-sided calcific tendinosis	1	1	2
3	None	3	2	4
4	None	3	1	4
5	None	3	1	4
6	None	0	1	1

*US*, ultrasound.

**Table 2:** Test characteristics of the supra-short ultrasound protocol for supraspinatus tears of the right shoulder, stratified by level of training.

Level of Training	Scans Completed	Sensitivity (95% CI)	Specificity (95% CI)
Emergency medicine residents	19	25.0% (0.6–80.6%)	80.0% (51.9–96.7%)
Ultrasound fellows	13	100% (29.2–100%)	90.0% (55.5–99.7%)
Attendings (without fellowship)	8	100% (15.8–100%)	100% (54.1–100%)
